# Supplementary material for: The transferability of lipid loci across African, Asian and European cohorts
Source: Nat Commun. 2019 Sep 24;10:4330. doi: 10.1038/s41467-019-12026-7 (PMC6760173; doi:10.1038/s41467-019-12026-7)
Supplement: Supplementary file 1 — Supplementary Information [file 41467_2019_12026_MOESM1_ESM.pdf]

"The transferability of lipid loci across African, Asian and European cohorts"

Kuchenbaecker et al.

Supplementary Information

Supplementary figures and tables

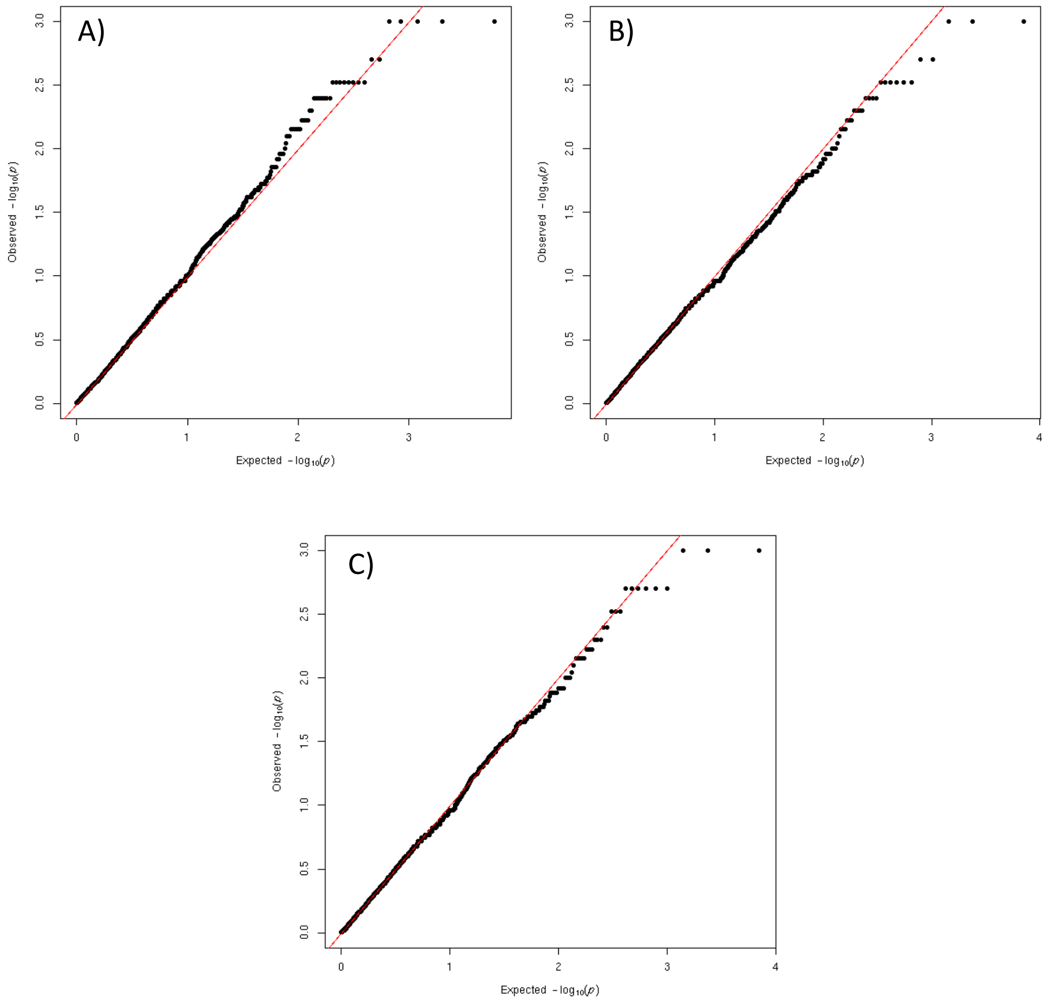

Supplementary Figure 1: QQ-plots for the p-values based on a permutation test from the trans-ethnic colocalization for simulated traits with distinct causal variants in UK Biobank and A) CKB and B) APCDR-Uganda C) UKHLS to provide an ancestry-matched control. Well-controlled type I errors manifest as a close match between the points and the diagonal line.

A)

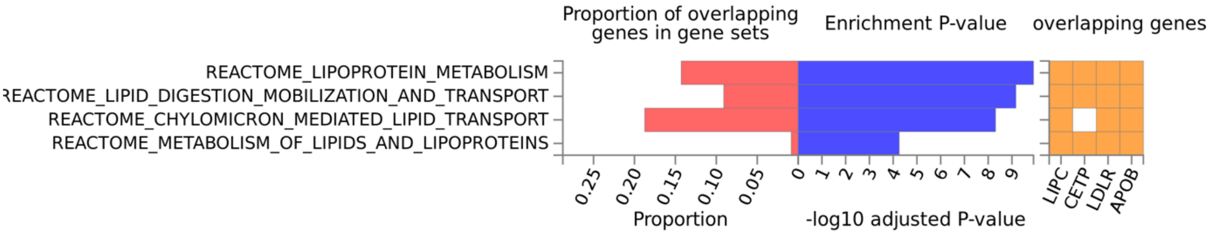

B)

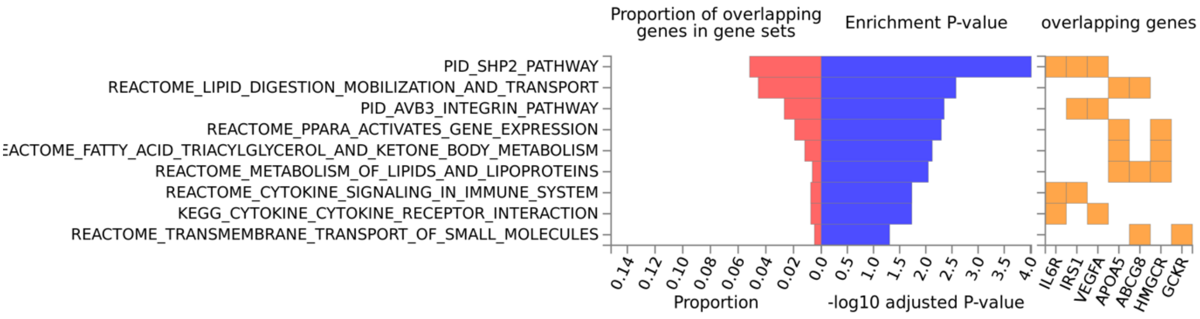

Supplementary Figure 2: Enrichment of canonical pathways (MsigDB c2) for genes proximal to established lipid-associated SNPs that A) replicate in APCDR-Uganda and B) do not replicate in APCDR-Uganda

A)

B)

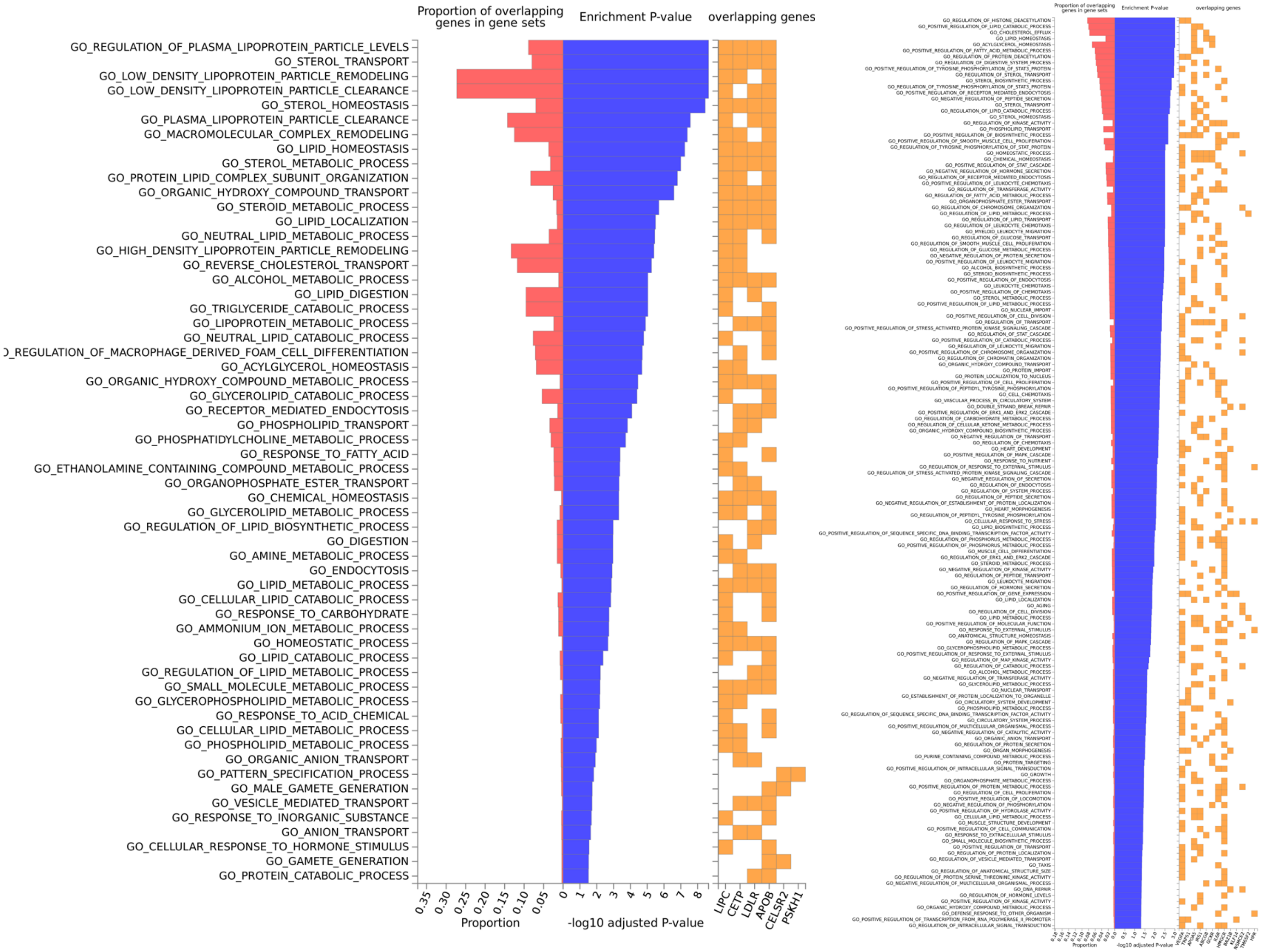

Proportion of overlapping genes in gene sets
Enrichment P-value
overlapping genes
GO_REGULATION_OF_PLASMA_LIPOPROTEIN_PARTICLE_LEVELS
GO_STEROL_TRANSPORT
GO_LOW_DENSITY_LIPOPROTEIN_PARTICLE_REMODELING
GO_LOW_DENSITY_LIPOPROTEIN_PARTICLE_CLEARANCE
GO_STEROL_HOMEOSTASIS
GO_PLASMA_LIPOPROTEIN_PARTICLE_CLEARANCE
GO_MACROMOLECULAR_COMPLEX_REMODELING
GO_LIPID_HOMEOSTASIS
GO_STEROL_METABOLIC_PROCESS
GO_PROTEIN_LIPID_COMPLEX_SUBUNIT_ORGANIZATION
GO_ORGANIC_HYDROXY_COMPOUND_TRANSPORT
GO_STEROID_METABOLIC_PROCESS
GO_LIPID_LOCALIZATION
GO_NEUTRAL_LIPID_METABOLIC_PROCESS
GO_HIGH_DENSITY_LIPOPROTEIN_PARTICLE_REMODELING
GO_REVERSE_CHOLESTEROL_TRANSPORT
GO_ALCOHOL_METABOLIC_PROCESS
GO_LIPID_DIGESTION
GO_TRIGLYCERIDE_CATABOLIC_PROCESS
GO_LIPOPROTEIN_METABOLIC_PROCESS
GO_NEUTRAL_LIPID_CATABOLIC_PROCESS
_REGULATION_OF_MACROPHAGE_DERIVED_FOAM_CELL_DIFFERENTIATION
GO_ACYLGLYCEROL_HOMEOSTASIS
GO_ORGANIC_HYDROXY_COMPOUND_METABOLIC_PROCESS
GO_GLYCEROLIPID_CATABOLIC_PROCESS
GO_RECEPTOR_MEDIATED_ENDOCYTOSIS
GO_PHOSPHOLIPID_TRANSPORT
GO_PHOSPHATIDYLCHOLINE_METABOLIC_PROCESS
GO_RESPONSE_TO_FATTY_ACID
GO_ETHANOLAMINE_CONTAINING_COMPOUND_METABOLIC_PROCESS
GO_ORGANOPHOSPHATE_ESTER_TRANSPORT
GO_CHEMICAL_HOMEOSTASIS
GO_GLYCEROLIPID_METABOLIC_PROCESS
GO_REGULATION_OF_LIPID_BIOSYNTHETIC_PROCESS
GO_DIGESTION
GO_AMINE_METABOLIC_PROCESS
GO_ENDOCYTOSIS
GO_LIPID_METABOLIC_PROCESS
GO_CELLULAR_LIPID_CATABOLIC_PROCESS
GO_RESPONSE_TO_CARBOHYDRATE
GO_AMMONIUM_ION_METABOLIC_PROCESS
GO_HOMEOSTATIC_PROCESS
GO_LIPID_CATABOLIC_PROCESS
GO_REGULATION_OF_LIPID_METABOLIC_PROCESS
GO_SMALL_MOLECULE_METABOLIC_PROCESS
GO_GLYCEROPHOSPHOLIPID_METABOLIC_PROCESS
GO_RESPONSE_TO_ACID_CHEMICAL
GO_CELLULAR_LIPID_METABOLIC_PROCESS
GO_PHOSPHOLIPID_METABOLIC_PROCESS
GO_ORGANIC_ANION_TRANSPORT
GO_PATTERN_SPECIFICATION_PROCESS
GO_MALE_GAMETE_GENERATION
GO_VESICLE_MEDIATED_TRANSPORT
GO_RESPONSE_TO_INORGANIC_SUBSTANCE
GO_ANION_TRANSPORT
GO_CELLULAR_RESPONSE_TO_HORMONE_STIMULUS
GO_GAMETE_GENERATION
GO_PROTEIN_CATABOLIC_PROCESS
0.35 0.30 0.25 0.20 0.15 0.10 0.05 0
Proportion
0 1 2 3 4 5 6 7 8
-log10 adjusted P-value
LIPC CETP LDLR APOB CELSR2 PSKH1

Supplementary Figure 3: Enrichment of GO biological processes (MsigDB c5) for genes proximal to established lipid-associated SNPs that A) replicate in APCDR-Uganda and B) do not replicate in APCDR-Uganda

Supplementary Table 1: Trans-ethnic genetic correlation estimates and p-values based on a Z-test whether the genetic correlation deviates from 1 for each lipid biomarker in one study with each lipid biomarkers in the other study

biomarker	correlation	standard error	p-value*
**GLGC2013 (European) – China Kadoorie Biobank**			
HDL-HDL	0.999	-	-
LDL-LDL	0.778	0.300	0.460
TG-TG	0.999	-	-
HDL - LDL	0.068	0.229	0.000
HDL - TG	-0.550	0.176	0.000
LDL - HDL	-0.238	0.143	0.000
LDL - TG	0.473	0.155	0.001
TG - HDL	-0.741	0.162	0.000
TG - LDL	-0.226	0.195	0.000
**GLGC2013 (European) – Biobank Japan**			
HDL-HDL	0.999	0.081	0.999
LDL-LDL	0.959	0.138	0.765
TG-TG	0.961	0.066	0.555
HDL - LDL	-0.055	0.097	0.000
HDL - TG	-0.592	0.130	0.000
LDL - HDL	-0.277	0.150	0.000
LDL - TG	0.294	0.056	0.000
TG - HDL	-0.481	0.176	0.000
TG - LDL	-0.038	0.091	0.000
**China Kadoorie Biobank – Biobank Japan**			
HDL-HDL	0.999	-	-
LDL-LDL	0.871	0.225	0.566
TG-TG	0.999	-	-
HDL - LDL	0.085	0.141	0.000
HDL - TG	-0.618	0.151	0.000
LDL - HDL	0.290	0.185	0.000
LDL - TG	0.180	0.169	0.000
TG - HDL	-0.862	0.186	0.000
TG - LDL	-0.097	0.153	0.000

* When the estimate is close to the boundary of 1, popcorn cannot compute the standard error and p-value

Supplementary Table 2: Associations of polygenic scores based on established lipid-associated loci and serum levels of each of the other lipid biomarkers in UKHLS, APCDR-Uganda, HELIC-MANOLIS, -Pomak, and CKB using a linear mixed model analysis with a score test to derive the p-value.

score - trait	correlation	SE (β)	p-value
**UKHLS**			
HDL - LDL	-0.0526	1.01E-02	2.02E-07
HDL - TG	-0.0720	1.02E-02	1.63E-12
LDL - HDL	-0.0516	1.01E-02	3.97E-07
LDL - TG	0.00845	1.02E-02	0.407
TG - HDL	-0.134	1.01E-02	1.67E-39
TG - LDL	0.0117	1.01E-02	0.247
**APCDR-Uganda**			
HDL - LDL	-0.0149	1.25E-02	0.234
HDL - TG	-0.0144	1.25E-02	0.249
LDL - HDL	-0.0773	1.25E-02	6.75E-10
LDL - TG	0.0422	1.25E-02	7.27E-04
TG - HDL	-0.00513	1.25E-02	0.681
TG - LDL	-0.0467	1.25E-02	1.86E-04
**HELIC-Pomak**			
HDL - LDL	-0.0722	3.09E-02	1.97E-02
HDL - TG	-0.128	3.10E-02	4.96E-05
LDL - HDL	-0.0608	3.18E-02	6.00E-02
LDL - TG	0.00343	3.19E-02	0.918
TG - HDL	-0.157	3.05E-02	6.16E-07
TG - LDL	0.0739	3.08E-02	1.70E-02
**HELIC-Manolis**			
HDL - LDL	0.00919	2.96E-02	0.756
HDL - TG	-0.0936	3.01E-02	2.13E-03
LDL - HDL	-0.0229	2.97E-02	0.442
LDL - TG	-0.00136	3.03E-02	0.964
TG - HDL	-0.133	2.98E-02	1.25E-05
TG - LDL	-0.00163	2.98E-02	0.956
**CKB**			
HDL - LDL	-0.0082	0.0183	0.65
HDL - TG	-0.0667	0.0200	8.6E-04
LDL - HDL	-0.0287	0.0186	0.12
LDL - TG	-0.0144	0.0203	0.65
TG - HDL	-0.0604	0.0183	9.8E-04
TG - LDL	-0.0499	0.0183	6.5E-03

Supplementary Table 3. Type I error rates from a simulation to assess the performance of trans-ethnic colocalization when causal variants are not shared. Phenotypes were simulated 10,000 times for each study and different values of effect sizes. Trans-ethnic colocalization was run to compare each to a reference set of 50,000 samples with British ancestry from UK Biobank. CKB was also down-sampled to match the sample size of APCDR-Uganda.

	Study (N)	APCDR-Uganda (4,597)	UKHLS (9,150)	CKB (72,473)	CKB (4,597)
**Beta**	0.10	0.044	0.043	0.065	0.059
	0.15	0.040	0.057	0.050	0.050
	0.20	0.048	0.059	0.073	0.059
	0.25	0.048	0.050	0.052	0.045

Supplementary Table 4. P-value for the trans-ethnic colocalization based on a permutation test for the JLIM model for established lipid-associated loci in UKHLS, China Kadoorie Biobank (CKB), Biobank Japan (BBJ) and APCDR-Uganda (UG).

				GLGC			reproducible[a]				JLIM p-value[b]		
**rs-id**	**chr**	**position**	**near gene**	**MAF**	**p-value**	**Multi[c]**	**UKHLS**	**CKB**	**BBJ**	**UG**	**CKB**	**BBJ**	**UG**
**HDL**													
rs4660293	1	40028180	PABPC4	0.21	6.1E-36	distant	uc	ns	cor	ns	0.8	0.014	NA
rs11755393	6	34824636	UHRF1BP1	0.36	4.2E-23	distant	ns	ns	cor	ns	0.035	0	NA
rs1178979	7	72856430	BAZ1B	0.18	1.3E-26	near	uc	uc	cor	ns	0	0	NA
rs4731702	7	130433384	KLF14	0.46	1.2E-35	distant	ns	cor	cor	ns	0.14	0.003	NA
rs4841132	8	9183596	PPP1R3B	0.9	1.0E-123	no	ns	ns	ns	cor	0.99	0.24	0.16
rs328	8	19819724	LPL	0.098	1.7E-316	near	cor	cor	cor	cor	0.21	0.005	0.62
rs2954033	8	126493746	NSMCE2	0.72	3.0E-61	near	cor	cor	cor	ns	0.94	0.002	NA
rs643531	9	15296034	TTC39B	0.88	3.8E-42	no	ns	ns	uc	uc	NA	NA	NA
rs2066714	9	107586753	ABCA1	0.15	3.6E-31	near	uc	cor	cor	uc	0.97	0.007	0.94
rs1883025	9	107664301	ABCA1	0.26	2.1E-118	near	uc	cor	cor	uc	0.84	0.8	0.97
rs2792751	10	113940329	GPAM	0.73	3.8E-21	near	ns	ns	cor	uc	0.002	0.002	NA
rs7350481	11	116586283	APOA5	0.91	3.2E-100	distant	cor	cor	cor	uc	0	0	0.99
rs964184	11	116648917	ZPR1	0.85	2.6E-217	near	cor	cor	cor	uc	1	1	1
rs10468017	15	58678512	LIPC	0.27	1.8E-306	near	cor	cor	cor	cor	1	0.98	0.99
rs1800588	15	58723675	LIPC	0.24	0	distant	cor	cor	cor	cor	0.007	0.009	0.017
rs247616	16	56989590	CETP	0.31	0	near	cor	cor	cor	cor	0	0	0
rs3764261	16	56993324	CETP	0.31	0	near	cor	cor	cor	cor	0	0	0
rs34065661	16	56995935	CETP	0.005	5.6E-103	near	uc	uc	uc	cor	0	0	0
rs16942887	16	67928042	PSKH1	0.13	9.8E-93	near	ns	ns	cor	cor	0.96	0.29	0.025
rs72836561	17	41926126	CD300LG	0.028	8.1E-111	no	uc	uc	uc	ns	NA	NA	NA
rs7241918	18	47160953	LIPG	0.85	1.2E-104	distant	cor	cor	cor	ns	0.16	1	1
rs116843064	19	8429323	ANGPTL4	0.02	4.8E-146	near	uc	ns	ns	uc	NA	NA	NA
rs769449	19	45410002	APOE	0.11	6.9E-129	near	cor	cor	cor	cor	0.009	0.02	0.95
rs386000	19	54792761	LILRB2	0.22	1.1E-41	distant	cor	uc	cor	cor	0	1	0.71
**LDL**													
rs11591147	1	55505647	PCSK9	0.015	0.0	near	uc	uc	uc	uc	0.94	0.64	0.92
rs12740374	1	109817590	CELSR2	0.22	0.0	near	cor	cor	cor	cor	0	0	0

rs1367117	2	21263900	APOB	0.28	3.6E-278	near	cor	cor	cor	cor	1	1	0
rs541041	2	21294975	APOB	0.81	1.3E-287	distant	cor	uc	uc	cor	1	1	NA
rs4245791	2	44074431	ABCG8	0.72	1.7E-120	near	uc	ns	ns	ns	NA	0.81	0.99
rs3846662	5	74651084	HMGCR	0.48	3.3E-128	near	cor	cor	cor	ns	0.002	0	1
rs2737229	8	116648565	TRPS1	0.34	8.9E-15	distant	cor	ns	cor	ns	0.015	0.025	NA
rs635634	9	136155000	IL6R	0.19	4.9E-109	near	ns	cor	cor	ns	0.96	0.97	NA
rs2000999	16	72108093	HPR	0.2	4.0E-71	distant	cor	cor	cor	ns	1	0	1
rs6511720	19	11202306	LDLR	0.11	0.0	near	cor	uc	uc	cor	0	NA	0
rs28399654	19	45316588	BCAM	0.027	7.5E-232	distant	cor	uc	uc	cor	1	1	NA
rs7412	19	45412079	APOE	0.075	0.0E+00	near	cor	cor	cor	cor	0	0	0
**Triglycerides**													
rs10889353	1	63118196	DOCK7	0.33	6.4E-170	no	cor	cor	cor	uc	0	0	0.88
rs676210	2	21231524	APOB	0.26	4.9E-118	near	cor	uc	cor	uc	1	1	1
rs1260326	2	27730940	GCKR	0.63	0.0	near	cor	cor	cor	ns	0	NA	NA
rs2943641	2	227093745	IRS1	0.66	4.9E-33	no	ns	ns	cor	ns	0.006	NA	1
rs6905288	6	43758873	VEGFA	0.59	9.0E-35	near	cor	ns	cor	ns	0	0	NA
rs1178979	7	72856430	BAZ1B	0.18	1.5E-179	near	cor	cor	cor	ns	0	0	NA
rs35332062	7	73012042	MLXIPL	0.12	5.2E-205	distant	cor	cor	cor	uc	0.99	1	NA
rs326	8	19819439	LPL	0.3	0.0	near	cor	cor	cor	uc	0.91	0.91	0.72
rs2954029	8	126490972	TRIB1	0.45	8.3E-205	near	cor	cor	cor	ns	0	0	1
rs1883025	9	107664301	ABCA1	0.26	1.2E-13	no	uc	ns	ns	ns	1	0.001	NA
rs7350481	11	116586283	APOA5	0.91	0.0	distant	cor	cor	cor	uc	0	0	0
rs11820589	11	116633862	APOA5	0.066	4.4E-133	near	cor	uc	uc	ns	0	1	1
rs2075291	11	116661392	APOA5	0.003	5.7E-65	near	uc	cor	cor	ns	NA	1	NA
rs10047462	11	116722041	SIK3	0.86	9.9E-180	near	cor	cor	cor	uc	0	0	1
rs247616	16	56989590	CETP	0.31	2.4E-38	near	ns	ns	cor	cor	0.014	0.024	0.78
rs116843064	19	8429323	ANGPTL4	0.02	4.2E-175	near	uc	ns	ns	ns	NA	NA	NA
rs58542926	19	19379549	TM6SF2	0.074	3.7E-125	no	cor	cor	cor	ns	NA	NA	NA
rs439401	19	45414451	APOE	0.63	2.7E-168	near	cor	cor	cor	uc	0.009	0.53	1

[a] indicates whether any variant from the credible set (“cor”) or any uncorrelated variant within 50kb (“uc”) is associated with the target biomarker at $p<10^{-3}$ in each of the target studies

[b] p-value from the JLIM trans-ethnic colocalization analysis using UKHLS as the comparison set

[c] indicates whether multiple independent hits have been reported within 50kb (“near”) or 1Mb (“distant”)

Supplementary Table 5: Summary of the genotyping, quality control, and imputation of each study

study	array	QC criteria SNPs	N SNPs genotyped	N SNPs after QC	Reference panel imputation	Imputation method	N SNPs imputed and QCed	QC criteria samples	N samples after QC
UK Household Longitudinal Study[1]	HumanCoreExome	Hardy-Weinberg equilibrium p-value < 1×10^{-4}, call rate < 98%, poor genotype clustering values (<0.4)	538,448	525,314	UK10K, 1000 Genomes v3	SHAPEIT, IMPUTE2	24,727,032	call rate <98%, autosomal heterozygosity outliers (>3SD), gender mismatches, duplicates (PI_HAT > 0.9), non-European ancestry	9,962 (9798 for HDL, 9797 for LDL, 9807 for TG)
African Partnership for Chronic Disease Research - Uganda[2]	HumanOmni2.5	call rate <0.97, Hardy-Weinberg equilibrium $p<10^{-8}$	2,369,382	2,330,014	1000G v3, 1,978 samples from Uganda	SHAPEIT, IMPUTE2	19,539,450	call rate <97%, heterozygosity (>3SD), gender mismatch, IBD>0.90, ancestry outliers (none)	6,407 (for all biomarkers)
China Kadoorie Biobank[3]	Custom Affymetrix Axiom Array	SNP call rate >0.98, plate effect $P>10^{-6}$, batch effect $P>10^{-6}$, HWE $P>10^{-6}$ (combined 10df χ^2 test from 10 regions), biallelic, MAF difference from 1KGP EAS < 0.2	701K/830K,	532,415	1000 Genomes v3	SHAPEIT3 and IMPUTE4	10,276,633	sample call rate >0.95, heterozygosity <mean+3SD, no chrXY aneuploidy, genetically-determined sex concordant with database	21,295 (20,810 for HDL, 17,662 for LDL, 20,219 for TG)

RIKEN Biobank Japan[4]	HumanOmniExpress	call rate < 0.99, minor allele frequency < 1%, Hardy–Weinberg equilibrium p≤ 1.0×10−6	~1M	NA	1000 Genomes v3 East Asians	MACH, minimac	6,108,953	call rate <0.98, closely related individuals based on IBD, non–East Asian outliers identified by PCA together with HapMap samples	162,255 (70,657 for HDL, 72,866 for LDL, 105,597 for TG)
Hellenic Isolated Cohorts[5,6] MANOLIS, Pomak	Whole-genome sequencing	VQSR with a tranche threshold of 99.4%, call rate <99%	NA	NA	NA	NA	24,163,896	sex checks, low concordance (π^<0:8) with chip data, duplicates, traces of contamination; checked but no exclusion necessary: depth, heterozygosity, transition/transversion (Ti/Tv) rate, missingness, ethnicity.	1,641 (1632 for HDL, 1630 for LDL, 1632 for TG), 1,945 (1915 for HDL, 1914 for LDL, 1916 for TG),
Global Lipids Genetics Consortium[7]	23 studies GWAS arrays, 37 Metabochip	study specific	196,710	study specific	HapMap	MACH	2.6M	study specific	188,577
Global Lipids Genetics	HumanExome		NA	242,289	NA	NA	NA	call rate, heterozygosity, sex discordance, GWAS discordance,	237,050

Consortium[8]								fingerprint concordance, PCA outliers	

Supplementary Table 6: Study description and mean levels of HDL-cholesterol, LDL-cholesterol and triglycerides (TG) in mmol/l

study	acronym	population	array	N SNPs genotyped, imputed	N samples	%female	mean age	mean HDL	mean LDL	mean TG
UK Household Longitudinal Study[1]	**UKHLS**	British	HumanCoreExome	248K, 26M	9,962	44	52	1.55	3.02	1.81
African Partnership for Chronic Disease Research - Uganda[2]	**APCDR-Uganda**	Ugandan	HumanOmni2.5	2.2M, 20M	6,407	56	34	1.02	2.05	1.18
China Kadoorie Biobank[3]	**CKB**	Chinese	Custom Affymetrix Axiom Array	701K/830K, 10M	21,295	62	60	1.37	2.19	1.69
RIKEN Biobank Japan[4]	**BBJ**	Japanese	HumanOmniExpress	6M	162,255	63	43	1.42	3.38	1.50
Hellenic Isolated Cohorts[5,6]	**HELIC-MANOLIS, -Pomak**	Isolated Greek populations	Whole-genome sequencing	24M	1,641, 1,945	42,34	62,45	1.28, 1.18	3.27, 3.09	1.61, 1.58
Global Lipids Genetics Consortium[7]	**GLGC2013** (meta-analysis)	European ancestry	23 studies GWAS arrays, 37 Metabochip	200K, 2.5M	188,577					
Global Lipids Genetics Consortium[8]	**GLGC2017** (meta-analysis)	European ancestry	HumanExome	242,289	237,050					

Supplementary Table 7: SNPs used to create genetic risk scores for HDL-cholesterol, LDL-cholesterol and triglycerides (TG) with reference alleles and weights.

HDL rs-id	reference	weight	LDL rs-id	reference	weight	TG rsid	reference	weight
rs11553746	C	0.015	rs13379043	T	-0.018	rs1011731	G	-0.015
rs2276853	G	-0.015	rs12748152	C	0.031	rs900399	A	-0.014
rs28932178	T	0.02	rs12740374	G	-0.16	rs10861661	A	0.019
rs740363	G	0.014	rs676210	G	-0.039	rs12748152	C	0.031
rs622082	A	-0.017	rs3756772	C	0.014	rs4846914	G	-0.039
rs2074158	T	-0.02	rs9376090	T	-0.025	rs676210	G	-0.071
rs1011731	G	0.015	rs4841132	A	0.057	rs13389219	C	-0.037
rs900399	A	0.019	rs2293889	T	-0.015	rs2943641	T	0.033
rs9816226	A	0.028	rs2954029	A	-0.048	rs13326165	A	0.02
rs12055786	C	-0.021	rs4149268	C	-0.015	rs9311651	A	-0.021
rs4871137	G	-0.022	rs687621	A	0.043	rs645040	G	0.023
rs10968576	A	-0.017	rs2068888	G	-0.016	rs442177	G	0.031
rs7076938	C	0.019	rs7941030	T	0.014	rs13133548	G	0.014
rs1037378	G	-0.015	rs173539	C	-0.033	rs9686661	C	0.042
rs746463	C	-0.017	rs9939224	T	-0.023	rs459193	A	0.023
rs7136716	A	0.021	rs7241918	G	0.02	rs998584	C	0.034
rs10861661	A	-0.017	rs6511720	G	-0.21	rs2745353	C	0.02
rs10483776	A	-0.02	rs7412	C	-0.54	rs4731702	C	-0.027
rs13379043	T	0.017	rs1132274	C	0.019	rs4841132	A	-0.035
rs8099014	C	0.015	rs4745	A	-0.015	rs1801177	G	0.17
rs2303108	T	-0.015	rs976002	A	0.023	rs2954029	A	-0.08
rs12748152	C	-0.043	rs13146272	C	-0.015	rs2068888	G	-0.032
rs4847399	G	-0.021	rs1016988	T	-0.02	rs2167079	C	-0.02
rs12740374	G	0.045	rs351855	G	-0.018	rs10892063	A	-0.058
rs12145743	T	0.017	rs3812594	G	-0.018	rs1106766	C	-0.03
rs4650994	G	-0.019	rs10885997	A	0.015	rs11057401	T	-0.028
rs1689800	A	-0.025	rs1891110	G	0.021	rs1800588	C	0.047
rs4846914	G	0.049	rs704	G	0.021	rs10468017	C	0.034
rs676210	G	0.06	rs2239619	C	0.018	rs1421085	T	0.019
rs2322659	T	-0.019	rs67710536	A	0.028	rs173539	C	-0.034
rs13389219	C	0.035	rs9646133	G	-0.019	rs9939224	T	-0.034
rs2943641	T	-0.036	rs11080150	A	-0.019	rs2925979	T	-0.029
rs2305637	C	-0.032	rs2125345	T	-0.024	rs2292642	C	-0.02
rs6762477	G	0.025	rs6062343	G	-0.014	rs489693	C	0.015
rs13326165	A	-0.025	rs4809330	A	-0.015	rs891088	A	-0.017
rs9311651	A	0.019	rs10903129	A	0.028	rs7255436	C	-0.019
rs645040	G	-0.021	rs11206510	T	-0.07	rs731839	G	-0.015
rs442177	G	-0.018	rs2479409	G	-0.047	rs7412	C	0.12
rs13133548	G	-0.017	rs505151	G	-0.09	rs7679	T	0.053

rs9686661	C	-0.032	rs10889353	A	-0.045	rs738322	A	-0.02
rs459193	A	-0.02	rs7515577	C	0.03	rs6062343	G	-0.018
rs34525648	G	-0.025	rs267733	A	-0.025	rs10889353	A	-0.077
rs11755393	A	-0.027	rs20558	T	0.015	rs541041	G	0.018
rs2894342	C	0.017	rs2738755	C	-0.015	rs1367117	G	0.023
rs998584	C	-0.026	rs541041	G	0.12	rs4245791	C	-0.019
rs35349911	C	-0.017	rs1367117	G	0.11	rs6882076	T	0.038
rs3756772	C	0.014	rs1801702	C	-0.091	rs1564348	T	0.02
rs2745353	C	-0.023	rs4245791	C	-0.072	rs7758229	G	0.018
rs9376090	T	-0.016	2:44066247	G	-0.11	rs4722551	T	-0.026
rs2303361	T	0.025	rs11556157	A	0.025	rs4921914	C	-0.035
rs4917014	T	0.017	rs2030746	C	0.014	rs2081687	T	-0.019
rs4731702	C	0.033	rs2287623	G	-0.021	rs1935	C	-0.029
rs3735080	C	-0.017	rs887829	C	-0.022	rs2255141	A	0.019
rs4841132	A	0.1	rs2290159	G	-0.021	rs2000999	G	0.021
rs1801177	G	-0.2	rs7640978	C	-0.033	rs11871606	C	0.016
rs2293889	T	0.029	rs2251219	T	0.016	rs58542926	C	-0.12
rs2954029	A	0.035	rs13315871	G	-0.038	rs157580	G	0.047
rs643531	C	0.053	rs3816873	T	-0.017	rs492602	A	0.018
rs4149268	C	-0.034	rs12654264	A	0.066	rs738409	C	-0.018
rs2066714	T	0.043	rs4530754	G	0.017	rs3769823	A	0.017
rs33918808	C	0.071	rs6882076	T	0.039	rs26008	T	-0.028
rs2230808	T	0.027	rs3757354	C	-0.033	rs3803357	C	-0.017
rs687621	A	0.015	rs1264562	G	0.015	rs7946	C	-0.016
rs970548	A	0.026	rs13192471	T	0.038	rs2785990	C	0.016
rs2068888	G	0.023	rs1055569	C	0.019	rs3947	G	0.024
rs2167079	C	0.041	rs1564348	T	0.047	rs3927680	T	-0.018
rs10838738	A	-0.032	rs7770628	C	-0.031	rs7901016	T	0.042
rs499974	C	-0.026	rs7758229	G	0.016	rs797486	C	0.02
rs10892063	A	-0.018	rs12670798	T	0.033	rs7157785	G	0.023
rs7941030	T	0.024	rs4722551	T	0.04	rs1077514	C	0.019
rs7134375	C	0.021	rs4921914	C	-0.022	rs6749689	T	-0.016
rs1106766	C	0.032	rs10102164	G	0.031	rs1049817	A	-0.056
rs7298565	G	0.03	rs2081687	T	-0.028	rs1344642	G	-0.015
rs11057401	T	0.033	rs2737229	A	-0.022	rs3748034	G	0.035
rs838880	C	-0.029	rs11136343	A	0.029	rs6831256	A	0.021
rs1800588	C	0.12	rs3780181	A	-0.037	rs16844401	G	0.03
rs10468017	C	0.11	rs1935	C	0.018	rs1126673	C	0.017
rs34317102	A	0.019	rs2255141	A	-0.028	rs4311394	A	0.018
rs1421085	T	-0.022	rs10128711	T	0.025	rs3873379	T	0.028
rs173539	C	0.23	rs11220462	G	0.043	rs2844480	C	0.023
rs9939224	T	0.2	rs11057830	G	0.023	rs1057373	C	0.03
rs5882	G	-0.092	rs4942486	T	-0.022	rs9271366	G	0.024

rs2925979	T	0.041	rs8017377	G	0.023	rs78957773	C	0.052
rs11869286	G	0.03	rs2000999	G	0.063	rs9472138	C	-0.02
rs2292642	C	0.028	rs34832584	G	0.02	rs4410790	T	0.015
rs7241918	G	0.077	rs314253	T	-0.02	rs2240466	G	-0.12
rs489693	C	-0.019	rs11871606	C	-0.027	rs38855	A	-0.014
rs891088	A	0.015	rs7188	A	0.048	rs11776767	G	0.022
rs2277998	G	0.016	rs11669576	G	0.058	rs326	A	-0.11
rs7255436	C	0.029	rs11557092	T	0.024	rs7940646	T	0.016
rs737337	T	-0.058	rs58542926	C	-0.1	rs174546	C	0.052
rs6511720	G	0.024	rs157580	G	0.072	rs12801636	G	-0.018
rs731839	G	0.017	rs1800437	G	-0.019	rs10047462	G	-0.11
rs2111504	T	0.02	rs492602	A	0.028	rs11820589	G	0.19
rs7412	C	0.098	rs364585	A	0.019	rs3135507	C	0.085
rs17695224	G	-0.028	rs7261862	T	-0.024	rs4149056	T	0.029
rs386000	G	0.054	rs6029526	T	0.035	rs7200543	A	0.024
rs12975366	T	-0.029	rs6016373	A	-0.024	rs12453522	A	0.021
rs1132274	C	-0.02	rs1053593	G	-0.016	rs12947658	A	-0.02
rs6120757	C	-0.017	rs738409	C	-0.018	rs7248104	G	-0.02
rs7679	T	-0.056	rs174546	C	-0.053	rs6818397	T	-0.021
rs181362	C	-0.028	rs2844529	G	0.018			
rs17738527	C	-0.018	rs1169288	A	0.037			
rs4823006	A	0.014						
rs738322	A	0.02						
rs138457	T	0.017						
rs267733	A	0.021						
rs1367117	G	-0.02						
rs2255141	A	-0.027						
rs11871606	C	-0.013						
rs157580	G	-0.026						
rs2785990	C	-0.015						
rs2240466	G	0.043						
rs326	A	0.11						
rs174546	C	-0.042						
rs10047462	G	0.023						
rs11820589	G	-0.087						
rs7200543	A	-0.019						
rs1997243	A	0.026						
rs8060686	T	0.056						

Supplementary references

1. Prins, B. P. *et al.* Genome-wide analysis of health-related biomarkers in the UK Household Longitudinal Study reveals novel associations. *Sci. Rep.* **7**, 11008 (2017).
2. Heckerman, D. *et al.* Linear mixed model for heritability estimation that explicitly addresses environmental variation. *Proc. Natl. Acad. Sci. U. S. A.* **113**, 7377–7382 (2016).
3. Chen, Z. *et al.* China Kadoorie Biobank of 0.5 million people: survey methods, baseline characteristics and long-term follow-up. *Int. J. Epidemiol.* **40**, 1652–1666 (2011).
4. Kanai, M. *et al.* Genetic analysis of quantitative traits in the Japanese population links cell types to complex human diseases. *Nat. Genet.* **50**, 390–400 (2018).
5. Gilly, A. *et al.* Cohort-wide deep whole genome sequencing and the allelic architecture of complex traits. *Nat. Commun.* **9**, 4674 (2018).
6. Gilly, A. *et al.* Very low depth whole genome sequencing in complex trait association studies. *Bioinforma. Oxf. Engl.* (2018). doi:10.1093/bioinformatics/bty1032
7. Global Lipids Genetics Consortium. Discovery and refinement of loci associated with lipid levels. *Nat. Genet.* **45**, 1274–1283 (2013).
8. Liu, D. J. *et al.* Exome-wide association study of plasma lipids in >300,000 individuals. *Nat. Genet.* **49**, 1758 (2017).
