# Supplementary material for: The transferability of lipid loci across African, Asian and European cohorts
Source: Nat Commun. 2019 Sep 24;10:4330. doi: 10.1038/s41467-019-12026-7 (PMC6760173; doi:10.1038/s41467-019-12026-7)
Supplement: Supplementary file 3 — Reporting Summary [file 41467_2019_12026_MOESM3_ESM.pdf]

nature research

Corresponding author(s): Karoline Kuchenbaecker

Last updated by author(s): Aug 15, 2019

Reporting Summary

Nature Research wishes to improve the reproducibility of the work that we publish. This form provides structure for consistency and transparency in reporting. For further information on Nature Research policies, see Authors & Referees and the Editorial Policy Checklist.

Statistics

For all statistical analyses, confirm that the following items are present in the figure legend, table legend, main text, or Methods section.

n/a	Confirmed	
☐	☒	The exact sample size (n) for each experimental group/condition, given as a discrete number and unit of measurement
☒	☐	A statement on whether measurements were taken from distinct samples or whether the same sample was measured repeatedly
☐	☒	The statistical test(s) used AND whether they are one- or two-sided<br>*Only common tests should be described solely by name; describe more complex techniques in the Methods section.*
☐	☒	A description of all covariates tested
☐	☒	A description of any assumptions or corrections, such as tests of normality and adjustment for multiple comparisons
☐	☒	A full description of the statistical parameters including central tendency (e.g. means) or other basic estimates (e.g. regression coefficient) AND variation (e.g. standard deviation) or associated estimates of uncertainty (e.g. confidence intervals)
☐	☒	For null hypothesis testing, the test statistic (e.g. F, t, r) with confidence intervals, effect sizes, degrees of freedom and P value noted<br>*Give P values as exact values whenever suitable.*
☒	☐	For Bayesian analysis, information on the choice of priors and Markov chain Monte Carlo settings
☒	☐	For hierarchical and complex designs, identification of the appropriate level for tests and full reporting of outcomes
☐	☒	Estimates of effect sizes (e.g. Cohen's d, Pearson's r), indicating how they were calculated

Our web collection on statistics for biologists contains articles on many of the points above.

Software and code

Policy information about availability of computer code

Data collection	Not applicable.
Data analysis	We used R v3.5.1, Popcorn v0.9.9, JLIM v1.0.2. Our trans-ethnic colocalization and simulation code is available on github

For manuscripts utilizing custom algorithms or software that are central to the research but not yet described in published literature, software must be made available to editors/reviewers. We strongly encourage code deposition in a community repository (e.g. GitHub). See the Nature Research guidelines for submitting code & software for further information.

Data

Policy information about availability of data

All manuscripts must include a data availability statement. This statement should provide the following information, where applicable:

- Accession codes, unique identifiers, or web links for publicly available datasets
- A list of figures that have associated raw data
- A description of any restrictions on data availability

The UKHLS EGA accession number is EGAD00010000918. Genotype-phenotype data access for UKHLS is available by application to Metadac (www.metadac.ac.uk). Summary statistics for GLGC (http://csg.sph.umich.edu/abecasis/public/) and Biobank Japan (http://jenger.riken.jp/en/) are publicly available. The HELIC genotype and WGS datasets have been deposited to the European Genome-phenome Archive (https://www.ebi.ac.uk/ega/home): EGAD00010000518; EGAD00010000522; EGAD00010000610; EGAD00001001636, EGAD00001001637. The APCDR committees are responsible for curation, storage, and sharing of the APCDR-Uganda data under managed access. The array and sequence data have been deposited at the European Genome-phenome Archive (EGA, http://www.ebi.ac.uk/ega/, study accession number EGAS00001000545, datasets EGAS00001001558 and EGAD00001001639 respectively) and can be requested through datasharing@sanger.ac.uk. Requests for access to phenotype data and summary statistics may be directed to data@apcdr.org. This is restricted to research-related purposes. Uploading and sharing of individual genetic data from CKB are subject to restrictions according to the Interim Measures for the Administration of Human Genetic Resources administered by the Human Genetic Resources Administration of China (HGRAC). Summary data including allele frequencies and GWAS summary statistics are available on application. This is restricted to research-related purposes. Other individual-level CKB data are available through www.ckbiobank.org, subject to

completion of a Material Transfer Agreement, either through Open Access or on application. CKB data access is subject to oversight by an independent Data Access Committee.

Field-specific reporting

Please select the one below that is the best fit for your research. If you are not sure, read the appropriate sections before making your selection.

☒ Life sciences ☐ Behavioural & social sciences ☐ Ecological, evolutionary & environmental sciences

For a reference copy of the document with all sections, see nature.com/documents/nr-reporting-summary-flat.pdf

Life sciences study design

All studies must disclose on these points even when the disclosure is negative.

Sample size	Sample size was was not predetermined. Analyses methods were adjusted to the N of each study. Genome-wide methods were applied to studies with >20,000 samples. We used polygenic scores for the others. The smallest non-European study had 6,407 samples which was well powered to identify PRS associations. When assessing locus replication we separated established major lipid loci with associations of p<10^-100 in the GLGC discovery sample in order to ensure sufficient power to see replication patterns.
Data exclusions	Each study carried out sample exclusions as part of their standard quality control. We describe the details in the supplement. We did not exclude any further samples.
Replication	Not available. However, we assess transferability of established loci, a form of replication in other populations.
Randomization	Not applicable.
Blinding	Not applicable.

Reporting for specific materials, systems and methods

We require information from authors about some types of materials, experimental systems and methods used in many studies. Here, indicate whether each material, system or method listed is relevant to your study. If you are not sure if a list item applies to your research, read the appropriate section before selecting a response.

Materials & experimental systems

n/a	Involved in the study	
☒	☐	Antibodies
☒	☐	Eukaryotic cell lines
☒	☐	Palaeontology
☒	☐	Animals and other organisms
☐	☒	Human research participants
☒	☐	Clinical data

Methods

n/a	Involved in the study	
☒	☐	ChIP-seq
☒	☐	Flow cytometry
☒	☐	MRI-based neuroimaging

Human research participants

Policy information about studies involving human research participants

Population characteristics	Biomarker levels were adjusted for age and gender. China Kadoorie Biobank also adjusted for fasting status, region and principle components.
Recruitment	These are secondary analyses. We provide references where the details of ethics are described.
Ethics oversight	These are secondary analyses. We provide references where the details of ethics are described.

Note that full information on the approval of the study protocol must also be provided in the manuscript.
